# Prognostic value of prostate circulating cells detection in prostate cancer patients: a prospective study

**DOI:** 10.1038/sj.bjc.6604912

**Published:** 2009-02-17

**Authors:** P Eschwège, S Moutereau, S Droupy, R Douard, J-L Gala, G Benoit, M Conti, P Manivet, S Loric

**Affiliations:** 1 Department of Urology, APHP Bicêtre University Hospital, Le Kremlin-Bicêtre, France; 2 Urology Unit, Antony Private Hospital, Antony, France; 3 INSERM U955EQ7, Department of Clinical Biochemistry & Genetics , APHP Mondor University Hospital, Créteil, France; 4 Department of General Surgery, APHP Cochin University Hospital, Paris, France; 5 Applied Molecular Technologies Center for Human Genetics, Saint-Luc University Hospital, Brussels, Belgium; 6 Laboratory of Clinical Biochemistry and Molecular Biology, APHP Lariboisière University Hospital, Paris, France

**Keywords:** prostatic neoplasms, neoplasm circulating cells, retropubic prostatectomy, surgery prognosis

## Abstract

In clinically organ-confined prostate cancer patients, bloodstream tumour cell dissemination generally occurs, and may be enhanced by surgical prostate manipulation. To evaluate cancer-cell seeding impact upon patient recurrence-free survival, 155 patients were prospectively enrolled then followed. Here, 57 patients presented blood prostate cell shedding preoperatively and intraoperatively (group I). Of the 98 preoperatively negative patients, 53 (54%) remained negative (group II) and 45 (46%) became intraoperatively positive (group III). Median biological and clinical recurrence-free time was far shorter in group I (36.2 months, *P*<0.0001) than in group II (69.6 months) but did not significantly differ in group II and III (69.6 months *vs* 65.0). Such 5-year follow-up data show that preoperative circulating prostate cells are an independent prognosis factor of recurrence. Moreover, tumour handling induces cancer-cell seeding but surgical blood dissemination does not accelerate cancer evolution.

In cancer surgery, local and distant recurrences may be explained by incomplete resection, leaving behind residual cancer cells in the resected stump area, and/or presence of undetectable regional micrometastases. Highly frequent detection of tumour-surrounding tissue-derived cells in the bloodstream during surgical tumour handling has often been reported in case of liver, colon, breast, gastric, and lung adenocarcinomas (Wong *et al*, 1999; Yamashita *et al*, 2000). Likewise, our previous study focusing on a potential risk for haematogenous spillage of prostate cancer cells during tumour handling has evidenced a 30–80% rise in the rate of circulating peripheral prostatic cells at the time of radical prostatectomy procedure during which the prostatic blood flows straight into the systemic circulation without any possibility of pedicle ligation (Eschwege *et al*, 1995). Among these circulating prostate cells, the presence of cancer cells with capabilities to further develop metastatic foci cannot be ruled out (Cristofanilli *et al*, 2004). Hence, additionally to the spontaneous prostate cell spillage inherent to the natural history of cancer, a mechanical spillage may occur during surgery. An unresolved issue, that seems important to surgeons, remains to know whether surgical manipulation of malignant tumours could promote haematogenous spread of tumour cells and further increase the incidence of distant metastasis. Thus, the aims of our prospective study were: (i) to assess the use of preoperative prostate cell detection as a prognosis marker of prostate cancer biological recurrence, and (ii) to elucidate whether tumour manipulation during retropubic radical prostatectomy (RP) may accelerate cancer dissemination.

## Materials and methods

We enrolled prospectively 176 consecutive men presenting biopsy-established prostate adenocarcinoma clinically confined to the prostate gland according to standard criteria and undergoing RP. None of them received hormonal therapy before surgery. Among this group, 21 were lost and only 155 patients were analysed. Clinical and pathological staging was performed using the TNM92 system, and patient stages were either pT2N0M0 or pT3N0M0 (casuistic is summarised in Table 1). Informed consent was obtained from each patient, and the study was approved by the ethical committee of our institution. Two 7 ml antecubital venous blood samples (EDTA-containing vacutainers, Beckton Dickinson, Le Pont de Claix, France) were drawn preoperatively 1 day before RP (referred to as preoperative sample) and, during surgery, 5 min after prostate removal (intraoperative sample). Haematogenous spread of prostate cells was assessed by a dual PSA/PSMA RT–PCR assay using very specific PSMA primers (forward PSMAF: gaatgccagagggcgatcta; reverse PSMAR: ttctgtgcatcatagtatcc; nested forward PSMANF: ggagtcattctctactccga; nested reverse PSMANR: ctctgcaattccacgcctat) and PSA (PSAF: tgcgcaagttcaccctca; PSAR: ccctctccttacttcatcc; PSANF: ctgtgtgctggacgcttg; PSANR: acctcacacctaaggaca) and considered positive when both markers were found to be expressed. Briefly, after RNA extraction and reverse transcription into cDNA as previously described (Eschwege *et al*, 1995; Tombal *et al*, 2003), a first row of PCR was performed using outer couples of PSA (PSAF, PSAR) and PSMA (PSMAF, PSMAR) primers in a multiplex fashion on 10 *μ*l cDNA (25 PCR cycles with 1 min hybridisation at 60°C at each cycle). After the first amplification was done, 1 *μ*l of the amplification product was poured into two distinct tubes to perform nested PCR using inner couples of primers (PSMANF, PSMANR in the PSMA tube, PSANF, PSANR in the PSA tube) for 25 additional cycles with 1 min hybridisation at 60°C at each cycle. As each forward primer was labelled at 5′-terminus by 6-FAM, fluorescent PCR products were analysed using Genescan technology on a 3100 genetic analyser (Applied Biosystems, Les Ulis, France). Robustness of the method was tested using LNCaP prostate cell line spiking experiments in normal blood. Limit of detection was found very low, detecting as few one prostate cell in 1 ml blood. Accuracy was also checked with intra- and inter-assay coefficients of variation of 8.9 and 10.5% respectively. No false positivity was observed in more than 100 healthy individuals who were tested as controls confirming the specificity of the method. Biochemical progression was defined as two consecutive serum PSA values of 0.2 ng ml^−1^ or greater (Elecsys2010, Roche, Meylan, France). To determine if RT–PCR results correlated with progression-free survival, a Cox regression was performed, and included preoperative PSA, Gleason score, pathological stage and RT–PCR results. The recurrence-free results were also assessed by the product-limit method of Kaplan–Meier. Comparisons between groups were performed by the log-rank method (SPSS10® and Prism4® statistical softwares).

## Results

**Figure 1:**
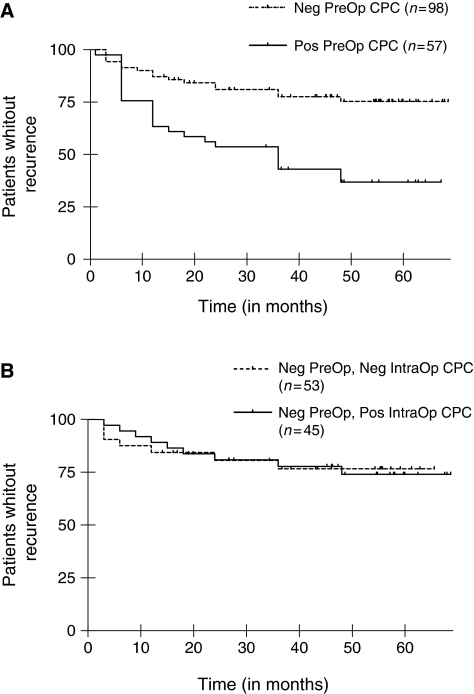
Progression-free survival for the three groups of patients. (**A**) Kaplan–Meier estimates of probabilities of progression-free survival. Patients with no preoperative circulating prostatic cells (Neg PreOp CPC) and with preoperative circulating prostatic cells (Pos PreOp CPC) are compared, *P*<0.0001 by the log-rank test, *χ*^2^=16.02. (**B**) Kaplan–Meier estimates of probabilities of progression-free survival. Patient without preoperative circulating prostatic cells and with (Neg PreOp, Pos IntraOp CPC) or without (Neg PreOp, Neg IntraOp CPC) intraoperative prostatic circulating cells are compared, *P*=0.818, NS by the log-rank test, *χ*^2^=0.053.

Blood dissemination of prostatic cells was preoperatively present in 57 patients (37%) (Group I) and absent in the 98 remaining patients (63%). Of these 98 preoperatively negative patients 45 (46%) became intraoperatively positive. Thus, regarding surgically induced blood prostatic cell shedding, two groups of patients can be differentiated: group II including preoperatively negative/intraoperatively negative (*n*=53) and group III preoperatively negative/intraoperatively positive patients (*n*=45). Although some of the clinical/biological factors remained relevant in the multivariate analysis (preoperative serum PSA, pathological stage, both *P*<0.01), the preoperative positivity of circulating prostate cells emerged as the strongest predictor of progression-free survival (*P*<0.001). The median biological and clinical recurrence-free time was two times as short for group I preoperatively positive patients as for groups II and III preoperatively negative patients (36.2 months, log-rank *χ*^2^=16.02, *P*<0.0001) (Figure 1A) thus showing that preoperative haematogenous spillage of prostate cells is adversely affecting prostate cancer patient survival. In contrast, the median recurrence-free time was not significantly different in group II when compared to group III (69.6 months and 65.0 months, respectively; log-rank, *χ*^2^=0.053, *P*=0.818, NS) (Figure 1B), revealing thereby that surgically induced prostate cell seeding in blood does not accelerate cancer evolution.

## Discussion

Refinements in surgical treatments have reduced surgical mortality in patients with solid epithelial tumours. The fate of cancer patients is generally linked to early tumour cell dissemination initially undetectable by conventional means (Cristofanilli *et al*, 2004). Blood-borne cancer cell detection arises as one of the recent methods to evaluate such seeding before any treatment. Our results unambiguously show the existing link between haematogenous spread of prostate cells, cancer recurrence and prognosis. Although, in our hand, PSA/PSMA transcripts specific RT–PCR quantification does not increase patient staging accuracy (data not shown), qualitative CPC detection (Yes/No answer on the basis of the presence of specific prostate transcripts in blood) appears to be sufficient to classify patients into two groups. The first one features patients with positive preoperative CPC detection (Group I) and a high probability of recurrence. The second one comprises patients with negative preoperative CPC detection (Group II+III) and a low probability of recurrence (Figure 1A). These data regarding clinically localised CaP patients complete the results of Moreno *et al* (2005) and more recently those of Okegawa *et al* (2008) that have shown correlation between CPC number and prostate cancer mortality in metastatic patients. Thus, as preoperative CPC detection or numeration seems a major indicator of primitive prostate tumour aggressiveness, it could be added to initial patient staging, to further propose a better treatment (either surgical or non-surgical).

Nevertheless, surgical cure requires that the tumour should be removed without inadvertent spillage of cancer cells at the time of organ handling and surgical manipulations. It is now well established that surgical variations do exist together with measurable rates of both local recurrence and survival (Birbeck *et al*, 2002). Patients who have persistently positive blood samples after radical surgery may be those at risk for systemic relapse but additional studies have also shown that most circulating tumour cells do not survive, with as little as 0.1% being responsible for the formation of secondary foci (Mareel *et al*, 1993).

Preliminary investigations with small numbers of samples have suggested that intraoperative tumour manipulations at surgery for primary breast, colorectal, and prostatic cancers induce tumour cell dissemination (Weitz *et al*, 1998; Weitz and Herfarth, 2001; Lintula *et al*, 2004). Nevertheless, in the absence of longer term follow-up, these studies failed to provide evidence that this cell dissemination may cause secondary metastatic deposits and affect survival.

Concern about dislodging of tumour emboli during carcinoma resection encouraged the introduction of the no-touch technique wherever primary lymphovascular pedicle ligation was possible. This surgical procedure was first assessed in colorectal carcinoma and, using molecular cell detection in a small subset of colorectal cancer patients, we evaluated the no-touch technique impact in colorectal surgery on intraoperative tumour cell dissemination (Sales *et al*, 1999). Nevertheless, in the only available prospective, randomised trial evaluating colorectal cancer patients, Wiggers *et al* (1988) failed to evidence any statistically significant impact of the no-touch technique on 5-year survival and suggested that surgically induced tumour cell dissemination did not affect prognosis. This is the reason why we were prompted to design a prospective study to evaluate prostate cancer cell dissemination at the time of radical prostatectomy procedure, which offers no opportunity to use the no-touch technique due to anatomic characteristics. For patients who did not undergo preoperative prostate cell dissemination, our 5-year follow-up results show no statistically significant differences between the absolute 5-year recurrence-free rates in intraoperative negative (group II) and intraoperative positive (group III) for prostatic cell shedding. Thus, we do evidence blood prostate cell spillage during surgical manipulation (45 patients among the 98 preoperatively negative were found positive intraoperatively). However, as no higher recurrence rate was observed in group III (intraoperatively positive) than in group II (intraoperatively negative), this surgical cell dissemination does not have any statistical consequences on the prognosis of these two preoperatively negative groups (II and III) (Figure 1B). Then, when we aggregate the comparative results for the three groups, it seems that the pejorative aspect of the prostate cell dissemination is a consequence of intrinsic cell characteristics (group I), and not a consequence of the surgical mechanical spreading of prostate cells where most of the cells are not yet competent to determine metastasis emergence (group III).

In radical surgery for prostate adenocarcinoma, haematogenous prostate cell spillage frequently occurs (i) preoperatively and (ii) during manipulation of the tumour. The preoperative blood spillage of prostatic cells show a strong biological recurrence predictive power, which is independent of other classical markers such as blood PSA initial levels. Thus, preoperative circulating prostate cells detection can be used as an additional marker to better stadify patients. Prostate cell blood dissemination was also detected intraoperatively, nevertheless, such spillage has not been shown to have any statistically significant adverse effect on recurrence, which seems to exclude tumour surgical management as a major cause of metastatic development. Our results suggest that intrinsic factors linked to the tumour itself mainly contribute to cancer evolution and subsequent poorer prognosis.

## Figures and Tables

**Table 1:** Clinical data for the three groups of patients

	**Group I**	**Group II**	**Group III**
Preoperative prostatic cell shedding	Yes	No	No
Intraoperative prostatic cell shedding	Yes	No	Yes
*n*	57	53	45
Age years (s.d.)	64.3 (4.2)	63.5 (5.1)	65.0 (4.9)
PSA ng ml^−1^ (s.d.)	10.9 (6.02)	9.29 (5.3)	10.5 (6.09)
Positive surgical margin	11	12	10
pTNM/UICC classification:			
pT2 N0 M0	23	22	14
pT3 N0 M0	18	16	18
Gleason score (s.d.)	6.78 (1.12)	6.39 (1.33)	6.58 (0.92)
